# Wildfire Smoke Classification Based on Synthetic Images and Pixel- and Feature-Level Domain Adaptation

**DOI:** 10.3390/s21237785

**Published:** 2021-11-23

**Authors:** Jun Mao, Change Zheng, Jiyan Yin, Ye Tian, Wenbin Cui

**Affiliations:** 1 School of Technology, Beijing Forestry University, Beijing 100083, China; MaoJun1997@bjfu.edu.cn (J.M.); tytoemail@bjfu.edu.cn (Y.T.); 2 Department of Fire Engineering, China Fire and Rescue Institute, Beijing 102202, China; jkldora@126.com; 3 Ontario Ministry of Northern Development, Mines, Natural Resources and Forestry, Sault St. Marie, ON 279541, Canada; Wenbin.cui@ontario.ca

**Keywords:** wildfire smoke classification, deep learning, synthetic images, adversarial training, domain adaptation

## Abstract

Training a deep learning-based classification model for early wildfire smoke images requires a large amount of rich data. However, due to the episodic nature of fire events, it is difficult to obtain wildfire smoke image data, and most of the samples in public datasets suffer from a lack of diversity. To address these issues, a method using synthetic images to train a deep learning classification model for real wildfire smoke was proposed in this paper. Firstly, we constructed a synthetic dataset by simulating a large amount of morphologically rich smoke in 3D modeling software and rendering the virtual smoke against many virtual wildland background images with rich environmental diversity. Secondly, to better use the synthetic data to train a wildfire smoke image classifier, we applied both pixel-level domain adaptation and feature-level domain adaptation. The CycleGAN-based pixel-level domain adaptation method for image translation was employed. On top of this, the feature-level domain adaptation method incorporated ADDA with DeepCORAL was adopted to further reduce the domain shift between the synthetic and real data. The proposed method was evaluated and compared on a test set of real wildfire smoke and achieved an accuracy of 97.39%. The method is applicable to wildfire smoke classification tasks based on RGB single-frame images and would also contribute to training image classification models without sufficient data.

## 1. Introduction

The frequency and size of wildfires have increased dramatically worldwide over the past few decades [1], posing a significant threat to natural resources while damaging the lives and property of individuals. Smoke detection can provide an earlier warning of a fire, as smoke usually appears earlier than flame can be detected in the early stages of a fire and is less likely to be obscured from view.

With the widespread use of deep convolutional neural networks in computer vision in recent years, more and more researchers have started to combine this method with smoke recognition tasks [2,3,4,5]. In deep learning-based smoke recognition, obtaining diverse wildfire smoke data as a positive sample is challenging due to the episodic nature of fire events, while it is relatively easy to collect forest environments as a negative sample. Under such conditions, the trained models are prone to false positive smoke detection, making it difficult to obtain satisfactory results. In addition, most of the visible images of wildfire smoke are acquired from RGB cameras carried by drones or webcams set up on lookouts. Therefore, the quality of the camera shots will greatly affect the quality of the raw image data, which will be worse if the lens becomes wet or dirty. In summary, acquiring a rich and diverse set of high-quality data is essential for deep learning-based smoke recognition tasks.

In order to increase the diversity of a dataset, the current mainstream approaches can be broadly divided into two categories, one based on GANs [6] to generate the data and the other using synthetic images to overcome the shortage of wildfire smoke data. GANs (generative adversarial networks) [6] have been used for data enhancement of wildfire smoke, which are trained with the idea of adversarial training and have achieved remarkable results in face generation and many other areas [7,8,9,10]. Namozov et al. [11] used a GAN network to generate fire images with winter and night backgrounds by the original images and added different seasons and times of the day. However, this method made it difficult to provide fire smoke with rich morphological variation, as the background of the original image and the shape of the fire smoke are retained during the generation process. Minsoo Park et al. [12] attempted to use CycleGAN [13] to learn a mapping from images of fire-free mountains to pictures of mountains with wildfires, thereby generating many pictures of wildfires. However, the class of fire-free mountain images used to train the model in this method is relatively limited. The missing alarm rate of smoke by the model trained on the generated data still needs to be further reduced. Previous approaches to generating wildfire image data based on GANs require a large amount of rich real wildfire image data or real wildland background image data as support, so the diversity of the generated data is largely limited by the diversity of the real data. This is the drawback of this approach.

There are two broad approaches using synthetic images to supplement the data, namely by generating smoke through indoor ignition experiments or 3D modeling software and then compositing the smoke image with the background image by linear overlay or direct rendering [14,15,16]. However, smoke and environment in ignition experiments differ significantly from that in wildland fires due to ignition environmental limitations. Therefore, in this study, we chose to simulate the smoke in 3D modeling software. However, as there is a difference in appearance between the synthetic smoke and the real smoke, this difference can cause the model to perform less well on real data. This problem arises when there is a difference in the statistical distribution between the synthetic training data and the real test data, i.e., a domain shift [17,18].

Researchers have proposed many domain adaptation methods to address the above-mentioned problem [19,20,21,22,23]. CycleGAN [13] is a representative type of pixel-level domain adaptation method, which transforms the source domain data in the original pixel space into a style in the target domain, capturing pixel-level and low-level domain shifts. However, using only pixel-level domain adaptation methods can result in the loss of high-level semantic features in the image during transformation. ADDA [24] is a classical feature-level domain adaptation method that aligns the features extracted by the network in the source and target domains, adapting high-level semantic features relevant to the specific task. Therefore, in this study, we used a combination of these two methods.

In this paper, a method was proposed to train deep convolutional neural networks for real smoke recognition through synthetic smoke data. Firstly, we used 3D modeling software to set various stochastic conditions to simulate virtual smoke, and then, the synthetic smoke image was obtained by rendering virtual smoke under virtual wildland background. After compression using the framework in [25], CycleGAN was used as the pixel-level domain adaptation to convert the synthetic smoke images into photorealistic smoke images in order to better solve the domain shift problem between the synthetic smoke and the real smoke. After this, all the data were split into a source and a target domain. The source domain data consisted of photorealistic smoke and real non-smoke, and the target domain data consisted of real smoke and real non-smoke. Following this, the domain-invariant features of the source and target domain data were learned by the feature adaptation: ADDA incorporated with DeepCORAL [26]. The proposed method is applicable to the task of wildfire smoke classification based on RGB single-frame images.

The full paper is structured as follows. We describe the building process of the synthetic smoke dataset, the CycleGAN-based pixel-level domain adaptation process, and the feature-level domain adaptation process based on ADDA with DeepCORAL in Section 2; Section 3 makes the experiment results using both models and the analysis of the experiments; and Section 4 makes the conclusion.

## 2. Materials and Methods

### 2.1. Data Collection

**Figure 1:**
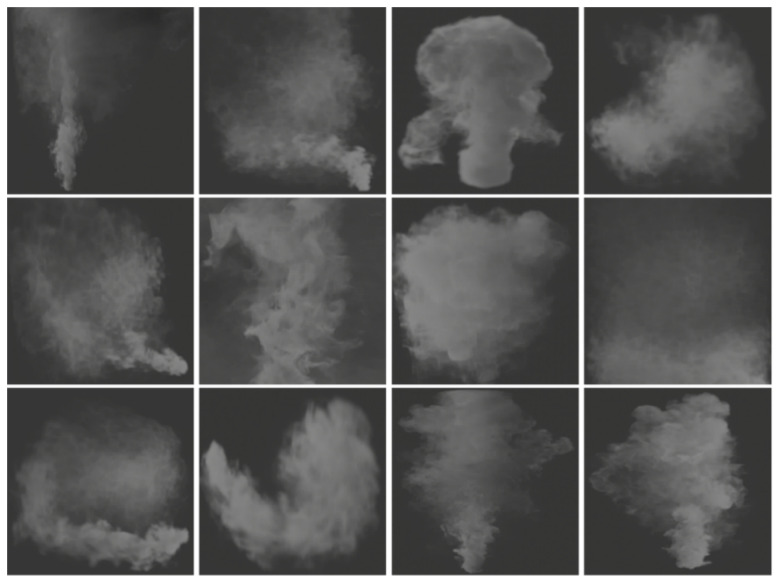
Sample images of virtual smoke.

Existing public datasets of forest fires tend to lack diversity [16]. In this paper, blender [27] was used to generate smoke with various appearances and resolutions by setting different lighting, wind, airflow, gravity, etc., and rendering the virtual smoke under any background image to produce synthetic images, as shown in Figure 1.

**Figure 2:**
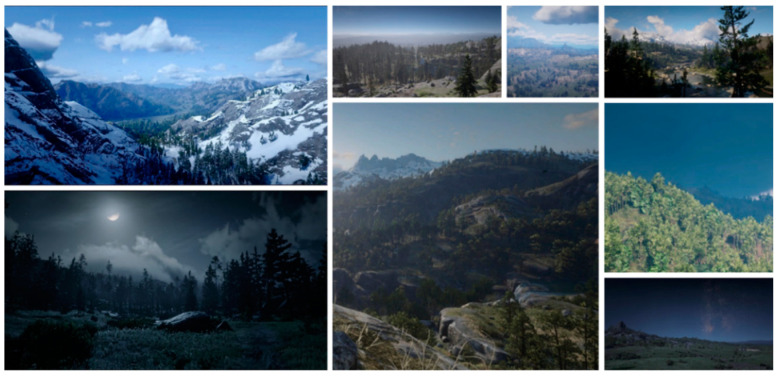
Sample images of virtual wildland background.

For the synthetic images suitable for subsequent pixel-level domain adaptation, the style of each image’s foreground and background should be uniform to ensure visual harmony. Therefore, virtual background images were collected in a video game, Red Dead Redemption 2, to match the virtual smoke images. Considering the diversity of the environment, we gathered 1500 images containing the scenes from different periods, different forest types, different environments, and different seasons. The samples of background image data are presented in Figure 2. 

**Figure 3:**
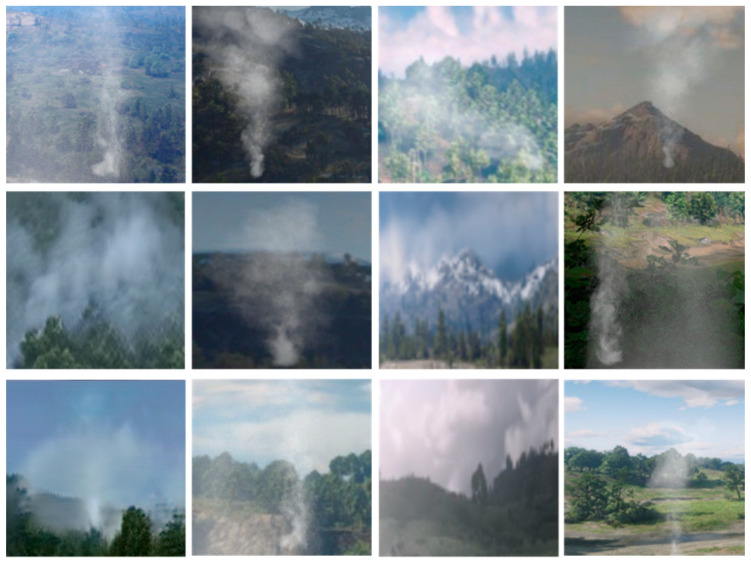
Samples of synthetic images.

We built a synthetic smoke dataset containing 2000 images of 256 × 256 size by rendering each virtual background image with a virtual smoke image. Figure 3 shows the sample of the synthetic image dataset.

**Figure 4:**
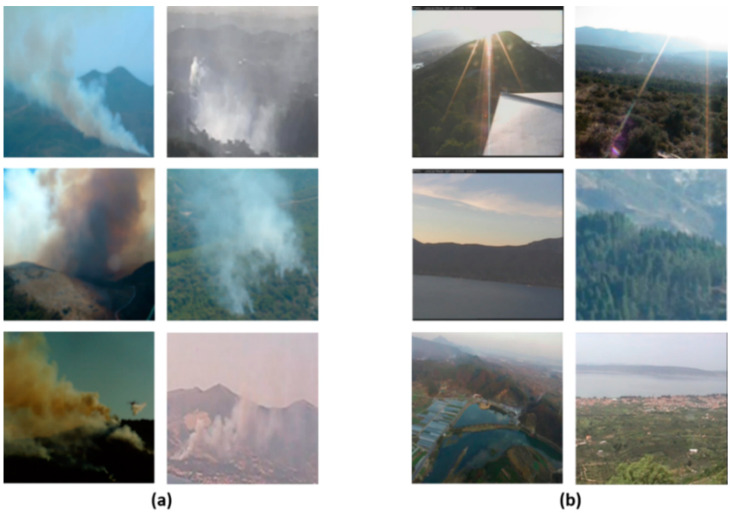
Samples of test images: (**a**) real smoke; (**b**) real non-smoke.

For real smoke data, wildfire smoke images were mainly collected from the internet, the public dataset from State Key Laboratory of Fire Science, USTC [28], and the public dataset from Yuan Feiniu [29]. After data cleaning, we obtained a total of 2500 images of wildfire smoke. In addition, a large number of real wildland background images were collected from the internet and selected, resulting in 4000 wildland background images. Of the images above, 1040 images were selected to build the test set—520 smoke and 520 non-smoke images—and Figure 4 shows some samples of the test set.

### 2.2. Pixel-Level Based Domain Adaptation

**Figure 5:**
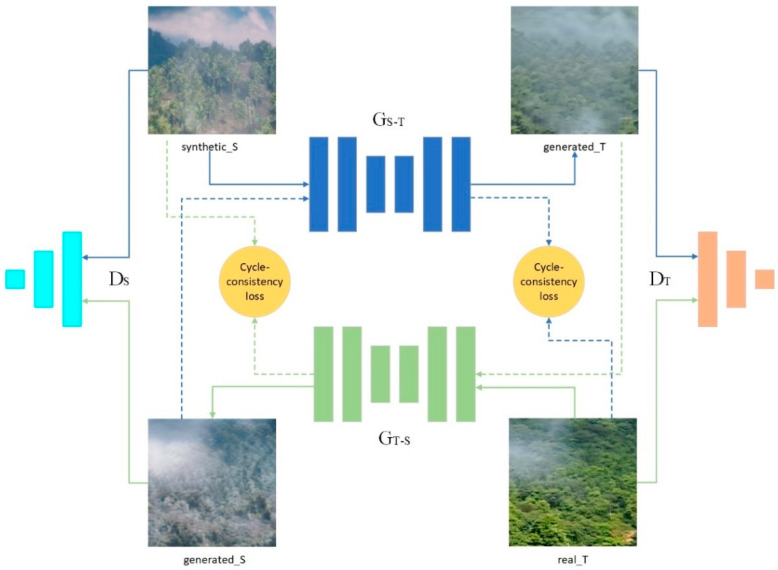
CycleGAN-based pixel-level domain adaptation architecture.

The pixel-level domain adaptation was made based on CycleGAN, as shown in Figure 5. synthetic_S is the source domain, and real_T is the target domain; the data $i_{S}$ in the source domain are synthetic smoke images, and the data $i_{T}$ in the target domain are real smoke images. The generator $G_{S-T}$ would learn a mapping function from the synthetic smoke images (the source domain) to the real smoke images (the target domain), the generator $G_{T-S}$, and vice versa. $G_{S-T}$ and $G_{T-S}$ are used to generate generated_T and generated_S, respectively. Our goal is to convert the source domain image into the style of the target domain image to obtain the data in generated_T, i.e., photorealistic smoke images. For mode collapse [6] and loss of structural information in the source domain images [30], the training process introduced cycle consistency loss to regularize. Specifically, for $i_{S}$ and $i_{T}$, one of our goals is $\;i_{S}\to G_{S-T}\left(i_{S}\right)\to G_{T-S}\left(G_{S-T}i_{S}\right)\approx i_{S}.$ The other goal is an inverse process for $i_{T}$. Equation (1) shows the whole cycle consistency loss:

(1) $$\begin{array}{c} {ℒ}_{cycle}\left(G_{S-T},G_{T-S},S,T\right)=E_{i_{S}\sim I_{S}}\left[\|G_{T-S}\left(G_{S-T}\left(i_{S}\right)\right)-i_{S}\|{}_{1}\right]+E_{i_{T}\sim I_{T}}\left[\|G_{S-T}\left(G_{T-S}\left(i_{T}\right)\right)-i_{T}\|{}_{1}\right] \end{array}$$

In addition, two discriminators $D_{S}$ and $D_{T}$ are trained to distinguish real and fake images. The adversarial loss [6] is formulated as Equations (2) and (3):

(2) $$\begin{array}{c} {ℒ}_{GAN}\left(D_{T},G_{S-T},S,T\right)=E_{i_{T}\sim I_{T}}\left[logD_{T}\left(i_{T}\right)\right]+E_{i_{S}\sim I_{S}}\left[\log\left(1-D_{T}\left(G_{S-T}\left(i_{S}\right)\right)\right)\right] \end{array}$$

(3) $$\begin{array}{c} {ℒ}_{GAN}\left(D_{S},G_{T-S},S,T\right)=E_{i_{S}\sim I_{S}}\left[logD_{S}\left(i_{S}\right)\right]+E_{i_{T}\sim I_{T}}\left[\log\left(1-D_{S}\left(G_{T-S}\left(i_{T}\right)\right)\right)\right] \end{array}$$

The overall loss of CycleGAN is defined as Equation (4):

(4) $$\begin{array}{c} {ℒ}_{CycleGAN}\left(G_{S-T},G_{T-S},D_{T},D_{S},S,T\right)={ℒ}_{GAN}\left(D_{T},G_{S-T},S,T\right)\\ +{ℒ}_{GAN}\left(D_{S},G_{T-S},S,T\right)\\ +\lambda {ℒ}_{cycle}\left(G_{S-T},G_{T-S},S,T\right). \end{array}$$

where λ is the weight of cycle consistency loss. To improve the training efficiency, the CycleGAN model was compressed using the general-purpose compression framework in this paper [25], reducing the computational cost of the generator and the model size.

### 2.3. Feature-Level Based Domain Adaptation

To learn high-level semantic information about the smoke images and further reduce the feature distribution difference between the photorealistic smoke images and the real smoke images, the feature-level domain adaptation method, which combined ADDA [24] with DeepCORAL [26], was proposed in this paper. In this section, the smoke images in the source domain are the photorealistic smoke images obtained by pixel-level domain adaptation, but all the non-smoke images in the source domain are real non-smoke images. This differs from the setting in traditional domain adaptation because the two categories in the source domain have different sources of data. However, our goal is to achieve the binary classification of smoke and non-smoke images, so we only need to focus on the generalized features of smoke and not too much on the features of non-smoke images. Experimentally, this setup did not affect the performance of the model. The images in the target domain are made up of real smoke images and real non-smoke images.

**Figure 6:**
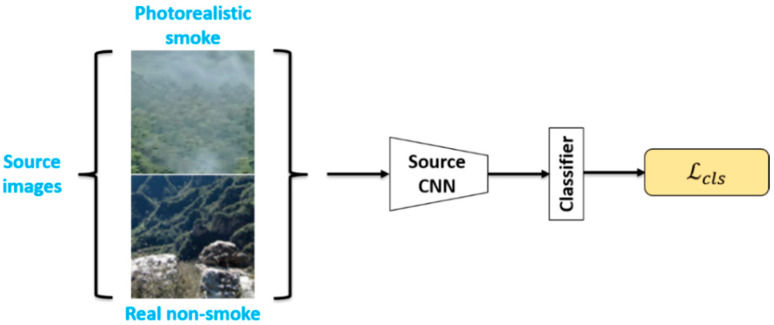
Pre-training phase for feature-level domain adaptation. The input source images in this phase are all labeled.

The source domain images $X_{S}$ are used with the label $Y_{S}$ throughout the feature-level domain adaptation process, while the target domain images $X_{T}$ are used without the label. The aim of feature-level domain adaptation is to train a target representation $M_{T}$ and classifier $C_{T}$ that can accurately classify the target images into two classes, including smoke and non-smoke, even in the absence of domain annotations. Since it is not possible to perform supervised training directly on the target domain, a source representation mapping $M_{S}$ and a source classifier $C_{S}$ were trained using the source domain images in the pre-training phase, as shown in Figure 6, where the source classifier is trained using the standard cross-entropy loss:

(5) $$\begin{array}{c} \underset{M_{S},C}{\min}\;{ℒ}_{cls}\left(X_{S},Y_{S}\right)=-E_{\left(x_{S}\;,\;\;y_{S}\right)\sim \left(X_{S}\;,\;Y_{S}\right)}\overset{K}{\underset{k=1}{\sum }}I_{\left[k=y_{S}\right]}logC\left(M_{S}\left(x_{S}\right)\right) \end{array}$$

In the adversarial adaptation phase, the main objective is to regularize the source mapping $M_{S}$ and the target mapping $M_{T}$ training, thus minimizing the feature distributions extracted by the source and target mappings: $M_{S}\left(X_{S}\right)$ and $M_{T}\left(X_{T}\right)$. Under such conditions, the source classifier $C_{S}$ can be used directly in the target representation, i.e., $C=C_{S}=C_{T}$.

**Figure 7:**
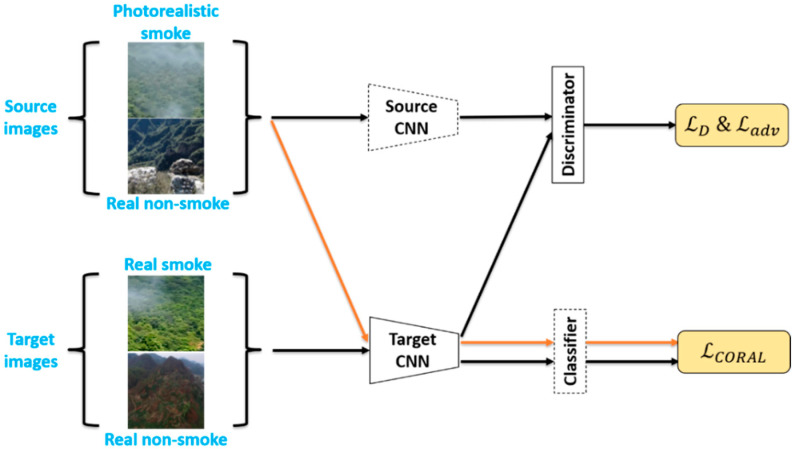
The adversarial adaptation phase of feature-level domain adaptation.

Based on the idea of adversarial training, the training losses of the domain classifier D and the target mapping $M_{T}$ are optimized in an alternating minimization process, as shown in Figure 7. First, for domain classifiers, the training goal is to accurately distinguish whether the data come from the source or target domain. The domain classifier D is, therefore, optimized using standard supervised loss, defined as follows:

(6) $$\begin{array}{c} \;\underset{D}{\min}\;{ℒ}_{D}\left(X_{S},X_{T},M_{S},M_{T}\right)=-E_{x_{S}\sim X_{S}}\left[logD\left(M_{S}\left(x_{S}\right)\right)\right]-E_{x_{T}\sim X_{T}}\left[\log\left(1-D\left(M_{T}\left(x_{T}\right)\right)\right)\right] \end{array}$$

When training the target mapping $M_{T}$, the standard cross-entropy loss is also used, as defined in Equation (7):

(7) $$\begin{array}{c} \underset{M_{T}}{\min}\;{ℒ}_{adv}\left(X_{S},X_{T},D\right)=-E_{x_{T}\sim X_{T}}\left[logD\left(M_{T}\left(x_{T}\right)\right)\right] \end{array}$$

In such a setting, the optimization of the target mapping $M_{T}$ and the domain classifier D is performed in adversarial training. In addition, to further align the correlation of features in the source and target domains, the calculation of the CORAL loss [26] was added to ADDA [24]. This is to align the second-order statistics of the source and target domain feature distributions, which helps to confuse the feature distributions. The definition of a CORAL loss is as follows:

(8) $$\begin{array}{c} {ℒ}_{CORAL}=\frac{1}{4d^{2}}\|CM_{S}-CM_{T}\|{}_{F}^{2} \end{array}$$

where $CM_{S}$ and $CM_{T}$ are the covariance matrices of d-dimensional features in the source domain and the target domain, respectively. The CORAL loss is also computed in the adversarial adaptation phase, as shown in Figure 7, where the learning rate of the classifier C was adjusted to 0 when performing backpropagation so that the CORAL loss only trains the target mapping $M_{T}$.

Therefore, the overall loss function of the target mapping $M_{T}$ is shown below:

(9) $$\begin{array}{c} ℒ={ℒ}_{adv}+\lambda {ℒ}_{CORAL} \end{array}$$

where λ denotes the weight of CORAL loss during training, which varies from 0 to 1 with training epochs.

In summary, the overall process of feature-level domain adaptation is as follows: first, a Source CNN and classifier are trained using the source domain images. After the training, the parameters of these two parts are no longer updated. In the adversarial training phase, the initial weights of the Target CNN are the same as those of the Source CNN, and the source and target domain images are used as inputs to the Source CNN and Target CNN, respectively. The features obtained after mapping are used to calculate ${ℒ}_{D}$ and $\;{ℒ}_{adv}$. At the same time, the source images and the target images are jointly used as input to the Target CNN, and the mapped features are then used as input to the pre-trained classifier, and the final output is used to calculate ${ℒ}_{CORAL}$. 

## 3. Experiments and Discussion

### 3.1. Dataset

For the pixel-level domain adaptation (PDA), 2000 synthetic smoke images and 1800 real smoke images were used, as shown in Table 1. After pixel-level domain adaptation, the 2000 synthetic smoke images were converted into photorealistic smoke images. The 2000 photorealistic smoke images were subjected to a series of data augmentations such as horizontal flip, gamma correction, color dithering, and contrast enhancement, and a total of 5000 images were selected as smoke samples in the source domain for feature-level domain adaptation (FDA). For smoke samples in the target domain, 5000 real smoke images were obtained by data augmentation of the original real wildfire smoke images. For both the non-smoke samples in the source and target domains in FDA, real wildland background images were used after data augmentation, resulting in 5000 real non-smoke images in the source domain and 5000 real non-smoke images in the target domain. The specific composition of the dataset is shown in Table 2. The test set included 520 real wildfire smoke images and 520 wildland non-smoke images, as shown in Table 3.

### 3.2. Implementation Details

In the PDA (pixel-level domain adaptation) phase, the batch size was set to 1. The Adam optimizer was used for training with an initial learning rate of 0.0002. After 100 epochs, the learning rate decayed linearly to zero in the following training process for another 100 epochs.

For FDA, the feature extraction part of ResNet-50 [31] was used as the network structure for Source CNN and Target CNN. As shown in Figure 6, first, the Adam optimizer was used for training in the pre-training phase, with the initial learning rate set to 0.0001. During the training process, the learning rate was adjusted every 10 epochs, with each learning rate being 0.5 times the previous one, for a total of 100 epochs.

As shown in Figure 7, in the adversarial training phase, the parameters of the pre-trained Source CNN were fixed and shared weights with the Target CNN and trained the Target CNN on this basis. The learning rate was set to 0.00001 for the Target CNN part, 0.0001 for the Discriminator, and 0 for the Classifier in order to keep the classifier parameters constant, thus allowing the CORAL loss to be used to train only the Target CNN. A total of 200 epochs were trained in this phase.

**Figure 8:**
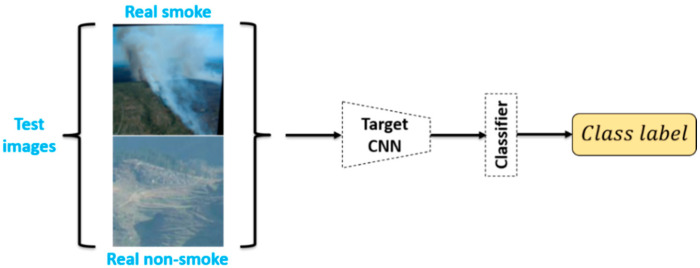
Test phase.

Lastly, we fixed the trained Target CNN and Classifier and tested the performance of the model with data from the test set, as shown in Figure 8.

### 3.3. Evaluation Metrics

To better measure the performance of the final trained model, we referred to the evaluation metrics defined in [16], namely CD (correct detection rate), ED (error detection rate), and MD (missed detection rate), where CD denotes the proportion of samples that were correctly predicted in the entire test set, ED denotes the proportion of non-smoke images in the samples that were predicted as smoke, and MD denotes the ratio of the number of samples that were incorrectly detected as non-smoke to the number of all non-smoke samples. These three metrics can better evaluate the classification effectiveness of the model on the test set and are defined as follows:

(10) $$\begin{array}{c} CD=\frac{N_{TP}+N_{TN}}{N_{TP}+N_{TN}+N_{FP}+N_{FN}}\times 100\% \end{array}$$

(11) $$\begin{array}{c} ED=\frac{N_{FP}}{N_{TP}+N_{FP}}\times 100\% \end{array}$$

(12) $$\begin{array}{c} MD=\frac{N_{FN}}{N_{TN}+N_{FP}}\times 100\% \end{array}$$

where $N_{TP}$ indicates the number of wildfire smoke images predicted to be wildfire smoke, $N_{TN}$ indicates the number of non-smoke images identified as non-smoke, $N_{FP}$ indicates the number of non-smoke images predicted to be wildfire smoke, and $N_{FN}$ indicates the number of wildfire smoke images identified as non-smoke. The overall performance evaluation will be carried out using the wildfire smoke and non-smoke test sets.

### 3.4. Results and Discussion

To verify and compare the effectiveness of each component of the domain adaptation method, ablation experiments were carried out. From the experimental results, we found that when training the ResNet-50-based classification model using only source images (synthetic smoke and real non-smoke) or target images (real smoke and real non-smoke) in Table 2, the CD on the test set was below 0.7000 and the MD was above 0.4500, which reflects serious overfitting of the model. Essentially, this overfitting may be due to the difference in distribution between the training data and the test data. However, when the domain adaptation method was applied to train the classification model, the results on the test set were improved.

When using only the pixel-level domain adaptation method, the CD can be boosted to 0.7042, but the ED and MD were still high. In contrast, as shown in Table 4, the feature-level domain adaptation completely outperformed pixel-level domain adaptation on this dataset. However, we found that performing pixel-level domain adaptation before feature-level domain adaptation helped improve model performance. The CD increased to 0.7918 when using only DeepCORAL for feature-level domain adaptation and to 0.8569 when using only ADDA for feature-level domain adaptation. In both cases, there was some increase in the ED and MD. This suggested that using either ADDA or DeepCORAL alone would degrade the performance of the model, but ADDA performed better in comparison. A combination of ADDA and DeepCORAL achieved better results. This suggested that adding the calculation of CORAL loss to the structure of ADDA was helpful to further confuse the distribution of features. Based on this, by combining pixel-level domain adaptation with full feature-level domain adaptation, the CD was increased to 0.9739, and both the ED and MD were reduced to below 0.0400.

**Figure 9:**
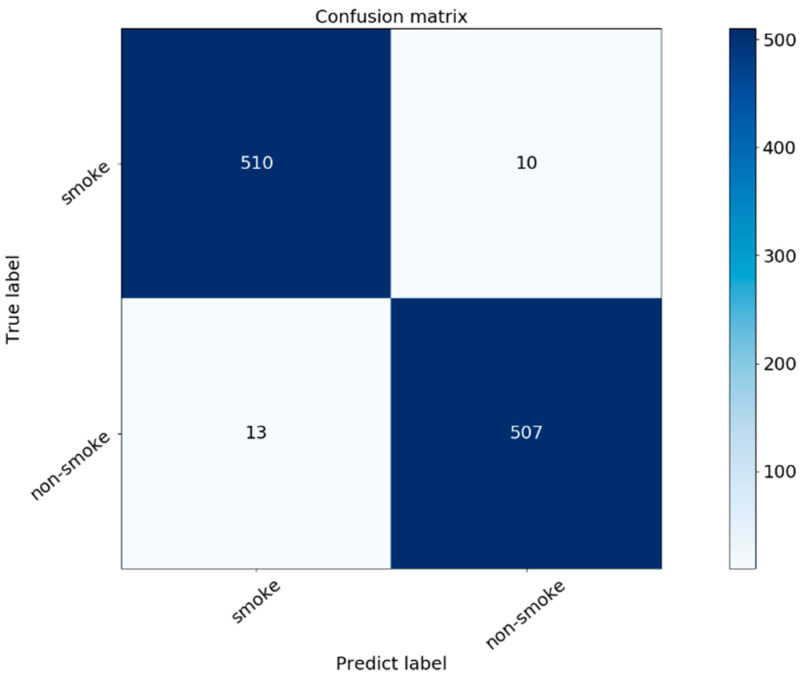
Confusion matrix of the proposed model.

In order to further evaluate the performance of the model proposed in this paper, a confusion matrix was used to represent the smoke classification ability of the model. Each column represents the predicted value, and each row represents the actual category. The confusion matrix of the model is shown in Figure 9.

**Figure 10:**
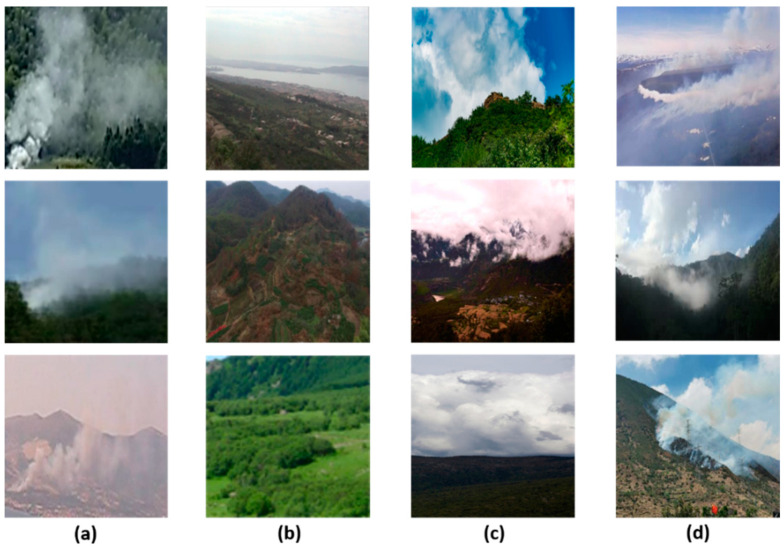
Samples of four cases: (**a**) true positive; (**b**) true negative; (**c**) false positive; (**d**) false negative.

In the confusion matrix, the squares at the top left and bottom right indicate the true positive and true negative cases, respectively, and the bottom left and top right indicate the false positive and false negative cases, respectively. Figure 10 shows a partial sample of each of these cases.

**Figure 11:**
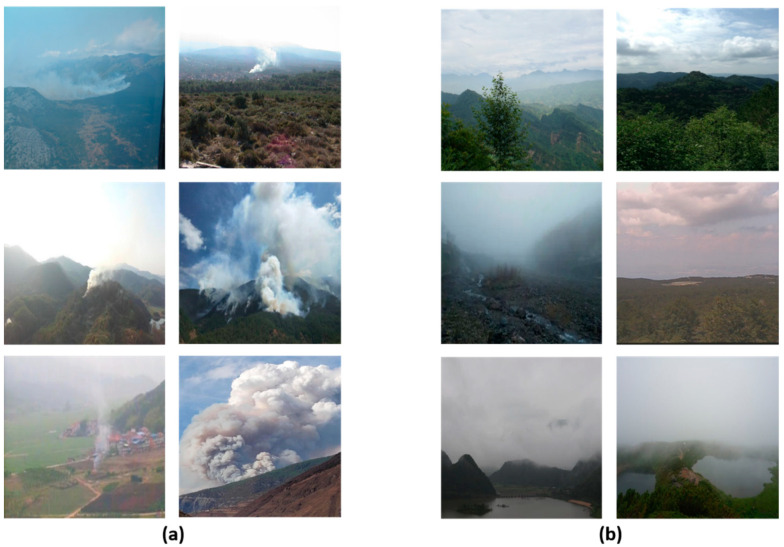
Examples of positive samples and negative samples: (**a**) wildland images of the cloud-smoke hybrid scenario; (**b**) non-smoke wildland images containing clouds or fog.

As can be seen by the samples in Figure 10c,d, false positive mostly occurs in exceptional weather in which the sky is heavily clouded or foggy, and false negative tends to occur when fire smoke overlaps with cloud cover or overly bright sky. However, the proposed method has been able to reduce the false positive rate and false negative rate more effectively in the above cases than models trained without deep domain adaptation. To demonstrate this, we collected 100 wildland images of the cloud-smoke hybrid scenario as positive samples and selected 100 non-smoke wildland images containing clouds or fog as negative samples, as shown in Figure 11. We used such data to test the performance of the proposed model in the cloud-smoke hybrid case and compared it with the model trained using only the target images in Table 2. The backbone of both models is still ResNet-50, and the comparison results are shown in Table 5. From the results, the proposed method improved the CD to 0.9382 and reduced both the ED and MD to below 0.0600 in the case of hybrid cloud-smoke. In summary, the proposed method can increase the recognition accuracy and reduce the false negative rate and false positive rate in the case of hybrid cloud-smoke.

To evaluate the recognition speed of the classification model, we calculated the average recognition time for test images on GPU and CPU, respectively, as shown in Table 6. The GPU is an NVIDIA GeForce RTX 2080 SUPER (NVIDIA, Santa Clara, CA, USA), and the CPU is an Intel Core i5-9600KF (Intel, Santa Clara, CA, USA). The single image recognition is faster on the GPU than on the CPU, and further improvements can be made by using better hardware or optimizing the network structure.

## 4. Conclusions

A new method was proposed to address single scenes and poor richness prevalent in samples from wildfire smoke image datasets. First, many morphologically rich smoke samples are simulated in 3D modeling software. These smoke samples are synthesized with a virtual 3D wildland background with rich environmental diversity, resulting in a synthetic wildfire smoke dataset. By successively performing domain adaptation in image pixel and feature spaces, the synthetic smoke dataset is better able to be used to train smoke classification models with higher generalization. The validity of the idea was experimentally verified, an accuracy of 97.39% was obtained on the test set, and the impact of different modules in the domain adaptation structure on performance was investigated.

The proposed method would be further extended to apply in image classification tasks with data shortage in various fields. In addition, we will try to train additional models with confusion-prone fire smoke targets and apply the idea of deep domain adaptation to the wildfire smoke object detection task, which will allow for the more accurate location of relatively small-scale smoke and also better alleviate the problem of poor recognition accuracy in the case of cloud-smoke hybrid.

## Figures and Tables

**Table 1:** Image datasets for pixel-level domain adaptation.

	Synthetic Images	Real Images
Smoke images	2000	1800

**Table 2:** Image datasets for feature-level domain adaptation.

	Smoke Images	Non-Smoke Images
Source images	5000	5000
Target images	5000	5000

**Table 3:** Testing set.

	Real Smoke Images	Real Non-Smoke Images
Test set	520	520

**Table 4:** Quantitative ablation results of the different domain adaptation architecture.

	CD	ED	MD
ResNet-50 w/source images	0.6348	0.3371	0.4712
ResNet-50 w/target images	0.6597	0.1988	0.5420
ResNet-50 w/PDA	0.7042	0.2989	0.2764
ResNet-50 w/FDA(only Deep CORAL)	0.7918	0.1053	0.1291
ResNet-50 w/FDA(only ADDA)	0.8569	0.1765	0.1138
ResNet-50 w/FDA(ADDA+DeepCORAL)	0.9242	0.0815	0.0655
ResNet-50 w/PDA+FDA(ADDA+DeepCORAL)	**0.9739**	**0.0386**	**0.0304**

Bold indicates that the results shown in this line are the best.

**Table 5:** Comparison of model performance in the cloud-smoke hybrid scenario.

	CD	ED	MD
ResNet-50	0.6598	0.2812	0.2157
ResNet-50 w/PDA+FDA(ADDA+DeepCORAL)	0.9382	0.0477	0.0534

**Table 6:** Average recognition time for a single image on different devices.

	Average Recognition Time (s)	Average Recognition Time (s)	Average Recognition Time (s)
GPU	0.0041	0.0039	0.0038
CPU	0.0595	0.0586	0.0580

## Data Availability

Publicly available datasets were analyzed in this study. The data can be found here: [http://smoke.ustc.edu.cn], [http://staff.ustc.edu.cn/~yfn/] (accessed on 1 November 2021).
